# Color Image Complexity versus Over-Segmentation: A Preliminary Study on the Correlation between Complexity Measures and Number of Segments

**DOI:** 10.3390/jimaging6040016

**Published:** 2020-03-30

**Authors:** Mihai Ivanovici, Radu-Mihai Coliban, Cosmin Hatfaludi, Irina Emilia Nicolae

**Affiliations:** Electronics and Computers Department, Transilvania University of Braşov, 500036 Braşov, Romania; coliban.radu@unitbv.ro (R.-M.C.); cosmin.hatfaludi@student.unitbv.ro (C.H.); irina.nicolae@unitbv.ro (I.E.N.)

**Keywords:** image complexity, color entropy, color fractal dimension, quasi-flat zones, JSEG

## Abstract

It is said that image segmentation is a very difficult or complex task. First of all, we emphasize the subtle difference between the notions of difficulty and complexity. Then, in this article, we focus on the question of how two widely used color image complexity measures correlate with the number of segments resulting in over-segmentation. We study the evolution of both the image complexity measures and number of segments as the image complexity is gradually decreased by means of low-pass filtering. In this way, we tackle the possibility of predicting the difficulty of color image segmentation based on image complexity measures. We analyze the complexity of images from the point of view of color entropy and color fractal dimension and for color fractal images and the Berkeley data set we correlate these two metrics with the segmentation results, more specifically the number of quasi-flat zones and the number of JSEG regions in the resulting segmentation map. We report on our experimental results and draw conclusions.

## 1. Introduction

By definition, image segmentation is the process of dividing an input image into regions called *segments* according to the application goal and specific criteria and choice of the parameter values. Segmentation is considered to be at the border between image processing and image analysis, as the segmentation map is often used for consequent object detection or recognition tasks. It is said that segmentation is one of the most difficult or complex operations performed on images [1]. What makes it difficult or complex? Is this difficulty or complexity strictly related to the segmentation approach or to the choice of parameters for an embraced segmentation approach? Does the complexity of the color image correlate with the number of segments in the segmentation map? In this article, we aim at bringing some arguments for the fore-mentioned questions and trying to answer the last one in an over-segmentation scenario. Over-segmentation still constitutes a current trend, dating back from the SuperPixels [2] and TurboPixels [3] approaches. The philosophy behind over-segmentation is to obtain a dense segmentation map which offers more flexibility to the consequent tasks, such as object detection and recognition [4].

First of all, we should recall the definitions of the two main concepts mentioned so far: difficulty and complexity, as very often we are tempted to consider that the two concepts are overlapping, or as exactly the same concept. However, they do not represent the same concept [5,6]: difficulty refers to the amount of effort needed to answer a question, address a problem or accomplish a task, while complexity refers to the kind of thinking, action or knowledge that is necessary for solving the same question, problem or task. In other words, a difficult task is hard to be performed, where a complex task refer to the degree of intricacy, entanglement, analysis, and evaluation which is required to solve the task. More specifically, a complex problem requires more creative and strategic thinking whereas difficultly relates to the effort or time necessary for solving the problem. In education and learning [7,8], difficulty and complexity are usually presented as completely orthogonal terms. In mathematics, higher difficulty can refer e.g., to higher precision for the floating points in a mathematical calculation, while higher complexity would refer to solving a linear equation system with complex numbers. In gaming, higher difficulty could mean e.g., when passing an obstacle requires more time or more player skills, while a higher number of rules or game elements that the player must understand and interact with, would refer to higher complexity. Considering an image segmentation scenario, the question is what can be viewed as complex and difficult, and if the two intersect or are orthogonal.

For color image segmentation, complexity could refer to the number of possible alternatives for solving the image segmentation problem, or strictly to the degree of entanglement within the approaches itself. In computer science, the complexity of an approach, more specifically of an algorithm for solving a specific problem, is expressed by the computational complexity or the *order of complexity* (the so-called *Big O notation* introduced by Paul Bachmann in 1894—see section 1.2.11 Asymptotic Representations in [9])—which indicates how many simple or elementary computations must be performed for solving the problem. For example, in order to apply a point-operator to an image of size M×N pixels, the order of complexity is O(M×N) as for each pixel it takes one computation; for a filtering operator with a filter mask of size m×n on the same image, the order of complexity is O(m×n×M×N). For pyramidal image segmentation approaches [10] the number of levels in the pyramid will increase the order of complexity proportionally. The order of complexity for image segmentation is very rarely reported by authors, but it is evident that active contours for image segmentation [11] exhibit a far more larger complexity compared to a contour extraction (edge detection) approach, for instance. For a given image segmentation approach, the difficulty could relate to the amount of contours or number of regions or the time necessary for the algorithm to accomplish the segmentation. Other researchers attempted to estimate the difficulty of image segmentation, as in [12] who uses different image features, including gray tone, color, gradient, and texture to predict the difficulty of a segmentation algorithm, considering the linear multiple regression prediction method. All the fore-mentioned image features are related to the image complexity itself. Consequently, image complexity impacts the segmentation difficulty—a complex image may be difficult to segment using a specific segmentation approach, either by taking more time to finish or by generating more segments.

The complexity of a color image may be defined in various ways. In [13] the complexity is defined as the effort of attention required for the act of perception. In [14] the complexity was defined as the irregularity of the arrangement for some binary patterns, heterogeneity of their elements, asymmetry and randomness. In [15] the link between the complexity of images and the measure of information, thus the entropy, is made. In [16] a complexity measure is proposed based on the number of components (lines, arches, objects) which required, in advance, the segmentation of the symbol/icon image of interest. In [17] the complexity is considered to be the amount of detail or intricacy of a symbol in a set of predefined symbols and icons. According to [18] the visual complexity of a color image could be a function of object variety or surface variety. More recently, in [19], the image complexity was modelled by using the independent component analysis (ICA), but ICA was used for the image entropy approximation. In [20] the image complexity is linked to the visual attention and based on maps generated by a computational model of human attention. In [21] the complexity of an image was linked to the fractal dimension. A particular set of approaches define image complexity based on image compression ratio, such as the approach presented in [22], where complexity is based either on the inverse of lossless compression ratio or on lossy compression and distortion. In [23] the complexity is defined as a linear combination of image features based on spatial, frequency and color properties and the optimal set of weights is determined using particle swarm optimization. In [24] three main factors that affect the human perception of visual complexity were identified to be the distribution of compositions, colors and contents and the authors designed 29 global, local and salient region features that represent those three factors.

In this article, we investigate the correlation between the intrinsic image complexity and the number of segments resulted in image over-segmentation. We also study the evolution of the chosen image complexity measures when the complexity of the color images is decreased by means of low-pass filtering. For our experiments we considered two types of images: color fractal images and natural images from the Berkeley image data base, as described later on. For the assessment of complexity of a color image, we embraced two widely used measures, the one of color entropy and the one of color fractal dimension. We considered for our experiments the over-segmentation approach entitled quasi-flat zones which is based on the concept of flat zones defined in [25] and the definition of connected components proposed in [26]. The motivation comes from the trend of using over-segmentation approaches, usually followed by a detection step based on machine learning approaches. A second image segmentation approach considered for our experiments was JSEG [27,28].

## 2. Materials and Methods

### 2.1. Image Data Bases

#### 2.1.1. Color Fractal Images

**Figure 1:**
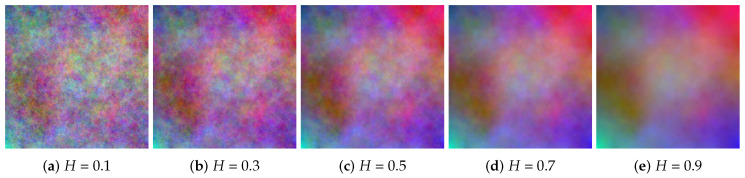
Color fractal images.

A subset of the color fractal image set we used is depicted in Figure 1. The images were generated using the midpoint displacement approach, with independent color components as described in [29]. The most complex image has a Hurst factor of 0.1, while the least complex image is obtained for H=0.9. The complete data set is available for download [30], along with the Matlab script for estimating the color fractal dimension, as described also in [29].

#### 2.1.2. Berkeley Segmentation Dataset

**Figure 2:**
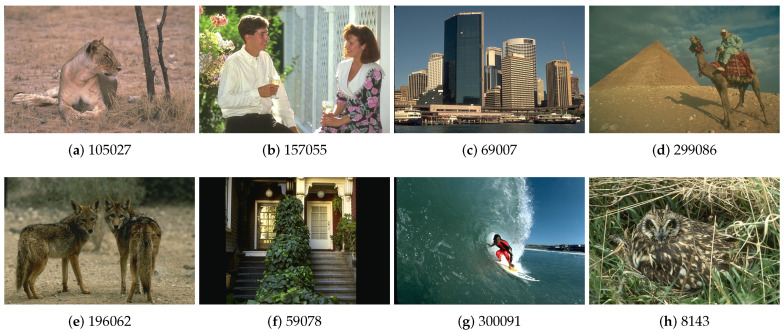
Examples of images from the BSDS500 data base.

The Berkeley Segmentation Dataset (BSDS500) [31] is a data base of natural images developed for research on image segmentation and boundary detection algorithms. The data base contains 500 color images of size 481 × 321 pixels, split into two groups: 300 images for training segmentation algorithms and 200 for testing. Each image has between four and seven associated reference segmentation maps. Figure 2 presents eight images from the data base.

### 2.2. Color Image Complexity

#### 2.2.1. Color Entropy

In information theory, Shannon [32] used the notion of entropy as a measure of the disorder in signals (see Equation (1) for a signal with *N* quantization levels, where $p_{i}$ is the probability of having a certain level present in the signal evolution). Based on this seminal definition, various other existing definitions developped: Rényi entropy (1961) was introduced as a generalization of the Shannon entropy (see Equation (2)), Hartley entropy, collision entropy and min-entropy, or the Kolmogorov entropy, which was presented in [33] as another generic definition of entropy.

(1) $$H=-\sum_{i=1}^{N}p_{i}\times \log_{2}\left(p_{i}\right)$$

(2) $$R=\frac{1}{1-\alpha }\log_{2}\left(\sum_{i}p_{i}^{\alpha }\right),\;\;\;\alpha \neq 1,\alpha >0$$

The same definition of Shannon entropy was embraced in [34] as one of his thirteen features proposed for texture characterization. The extension to multidimensional case of Shannon definition of entropy is straightforward, and that question was addressed in [35]. In the same paper, the limitations of both entropy and the current probabilistic box-counting fractal dimension estimator were shown, which lead to a saturation of the measure for the most complex color fractal images. In addition, from the definition of entropy itself it is known that the entropy can only underestimate the complexity of the object, i.e., the color texture in our case, by not taking into account the spatial arrangement of pixels.

#### 2.2.2. Color Fractal Dimension

Multi-scale approaches or measures are used for the analysis of color texture images. The most representative is the fractal dimension from fractal geometry [36]. Fractals are self-similar objects, independent of scale. Fractal dimension, the fundamental measure in fractal geometry, was defined to assess the complexity of fractal objects. The fractal or similarity dimension is a quantitative measure of the variations, irregularities or *wiggliness* of a fractal object [37]. The fractal dimension of an object is comprised in the interval [E,E+1], where *E* is the topological dimension of that object. In practice, the fractal dimension has been used for the discrimination between various signals or patterns exhibiting fractal properties, such as textures [38]. Often, the application of texture classification is image segmentation [1].

The theoretical fractal dimension is the Hausdorff dimension [39], also called Hausdorff-Besicovitch dimension. Because of its definition for continuous objects and its intrinsic complexity, the Hausdorff dimension is not used in practice, but equivalent fractal dimension estimates were defined and used. There are various approaches for the estimation of fractal dimension for digital signals/images exhibiting the property of self-similarity. The now classical approaches include: the probability measure [40,41], the Minkowski–Bouligand dimension, also known as Minkowski dimension or box-counting dimension [39], the δ-parallel body method (also called covering-blanket approach), morphological covers or Minkowski sausage [42], the gliding box-counting algorithm based on the box-counting approach [43] etc.

Several attempts were made to extend the fractal dimension estimation techniques to the color image domain. The initial approaches which link the fractal measures to colour images were marginal colour analysis [44]. The probabilistic box-counting approach was extended for the assessment of the complexity of color fractal images with independent color components and its validity was proved both mathematically and experimentally [29]. To the best of our knowledge, it was the first fully vectorial approach for estimating the probabilistic box-counting fractal dimension for color digital images. However, in [35] the limitations of this approach in estimating the color fractal dimension for the very high complexity color fractal images were emphasized. Other attempts in defining the fractal dimension for color images do exist. In [45] authors propose an approach inspired from the box counting paradigm, by dividing the image in non-overlapping blocks and defining the counting in the RGB color domain, for both synthetic and natural images. In [46,47] extensions of the differential box counting approach are proposed for color images in RGB color space. The experiments were performed both on generated images or images from the colored Brodatz texture data base (https://multibandtexture.recherche.usherbrooke.ca/colored_brodatz.html).

### 2.3. Color Image Segmentation Approaches

#### 2.3.1. Quasi-Flat Zones

Quasi-flat zones (QFZ) are morphological segmentation operators derived from the concept of flat zones [25]. Flat zones represent connected sets of pixels with the same gray value; this restrictive connectivity relation usually produces an extreme over-segmentation of the image. As a result, various definitions that use more relaxed connectivity rules have been introduced [26].

For instance, in the case of gray-scale images, the α-connectivity rule is defined as follows: two pixels are connected if there is a path of pixels linking them such that the difference between the values of two successive pixels is smaller than the local threshold value α. Thus, the definition for the QFZ of type $C^{\alpha }$ containing the pixel *p* from the gray-scale image *f* is [26]:

(3) $$\begin{array}{cc} C^{\alpha }\left(p\right) & =\left\{p\right\}\cup \left\{q\right|\exists a\mathrm{path}P=\left(p=p_{1},\ldots ,p_{n}=q\right),n>1,\\ & \mathrm{such}\mathrm{that}|f\left(p_{i}\right)-f\left(p_{i+1}\right)\left|\leq \alpha ,\forall 1\leq i<n\right\} \end{array}$$

$C^{\alpha }$ generates a unique partitioning of the image for a value of α, due to the equivalence relation imposed on the set of image pixels. An important issue with the $C^{\alpha }$ definition is the under-segmentation of the image, which can result even when using small values of α. As a consequence, several QFZ definitions based on supplementary connectivity rules have been developed, such as QFZs of the type $C^{\alpha ,\omega }$, which besides the local threshold α, also use a global threshold ω [26]:

(4) $$C^{\alpha ,\omega }\left(p\right)=⋁\left\{C^{\alpha^{\prime }}\left(p\right)|\alpha^{\prime }\leq \alpha \mathrm{and}R\left(C^{\alpha^{\prime }}\left(p\right)\right)\leq \omega \right\}$$

where the range function *R* computes the difference between the maximal and minimal values of the set given as its argument.

A straightforward strategy for extending the definition of $C^{\alpha ,\omega }$ QFZs to color images, based on the definition in Equation (4), is also given in [26]:

(5) $$C^{\alpha ,\omega }\left(p\right)=⋁\left\{C^{\alpha^{\prime }}\left(p\right)|\alpha^{\prime }\leq \alpha \mathrm{and}R\left(C^{\alpha^{\prime }}\left(p\right)\right)\leq \omega \right\}$$

where in this instance, α and ω are *vector* parameters and “≤” is the marginal ordering of vectors:

(6) $$\forall v,v^{\prime }\in R^{n},v\leq v^{\prime }\Leftrightarrow \forall i\in \left\{1,\ldots ,n\right\},v_{i}\leq v_{i}^{\prime }$$

The possible values of the parameters are restricted to vectors with the same value across all dimensions (for instance, for color RGB images: [0,0,0], [1,1,1],…). All the definitions presented above still induce, in most cases, some over-segmentation of the image [48].

#### 2.3.2. JSEG

The JSEG color image segmentation approach proposed by Deng [27,28] uses the J-factor as a criterion for the identification of heterogeneity zones. The J-factor was defined as a measure of local similarity between colors within an analysis window. The aim is to identify region homogeneity/heterogeneity considering a color texture perspective, based on the assumption that the color information in a region of the image can be reduced to the information provided by few representative colors. The J-factor represents the normalized variance of spatial distances with respect to class centers, given that image colors are classified.

For a set *Q* of *N* pixel locations $P_{i}$, the mean position *m* of all pixels is computed as $m=\frac{1}{N}\sum_{i=1}^{N}P_{i}$. If *Q* is classified into *C* classes $Q_{i}$ based on the color values at those locations, then $m_{i}$ be the mean position of the $N_{i}$ points of class $Q_{i}$: $m_{i}=\frac{1}{N_{i}}\sum_{P_{i}\in Q_{i}}P_{i}$. Then the total spatial variance is defined as $S_{T}=\sum_{q\in Q}||q-{m||}^{2}$, and the spatial variance relative to the $Q_{i}$ classes as $S_{W}=\sum_{i=1}^{C}S_{i}=\sum_{i=1}^{C}\sum_{q\in Q}||q-m_{i}{\left|\right|}^{2}$, the measure *J* is defined as:

(7) $$J=\frac{S_{B}}{S_{W}}=\frac{S_{T}-S_{W}}{S_{W}}$$

where *J* basically measures the distances between various classes $S_{B}$ over the distances between the members within each class $S_{W}$: a high value of *J* indicates that the classes are more separated from each other and the members within each class are closer to each other, and vice versa. Consequently, the resulting J-image is a gray-scale pseudo-image whose pixel values are the *J* values calculated over local windows centered on each pixel position. The higher the *J* value is, the more likely that the pixel is close to a region boundary.

First of all, an adaptive color quantization step is performed in order to reduce the number of colors in the image, which influences the segmentation result. The segmentation approach continues with a computation of the *J*-image at various scales, followed by the segmentation of the *J*-image starting at the coarsest level. Relatively uniform regions are identified by low *J* values. A region growing step is then performed, starting from the areas around *valleys*, which are local minima in the pseudo *J* image. The *J* values are averaged in the unsegmented part of a region, then the pixels with values below this average are included in the growing region. If a growing region is adjacent to one and only one valley, it is assigned to that valley. The process is iteratively repeated for the lower scales, ensuring that the segmentation approach determines the final localization of contours. The result of this iterative scheme is an oversegmented image and usually a region fusion step is performed to reduce this undesired effect.

## 3. Experimental Results

We performed a set of initial experiments with a set of nine images representing synthetic color fractal textures of varying complexity as a function of the Hurst parameter *H* (small *H* values are linked to high complexity and vice-versa). We chose this specific type of images as they do not exhibit any particular content, but instead a noise with properties of statistical self-similarity. For color image complexity we embraced the color entropy (CE) and the color fractal dimension (CFD). The chosen segmentation approaches were quasi-flat zones (QFZs) and JSEG, which were all detailed in the previous section.

**Figure 3:**
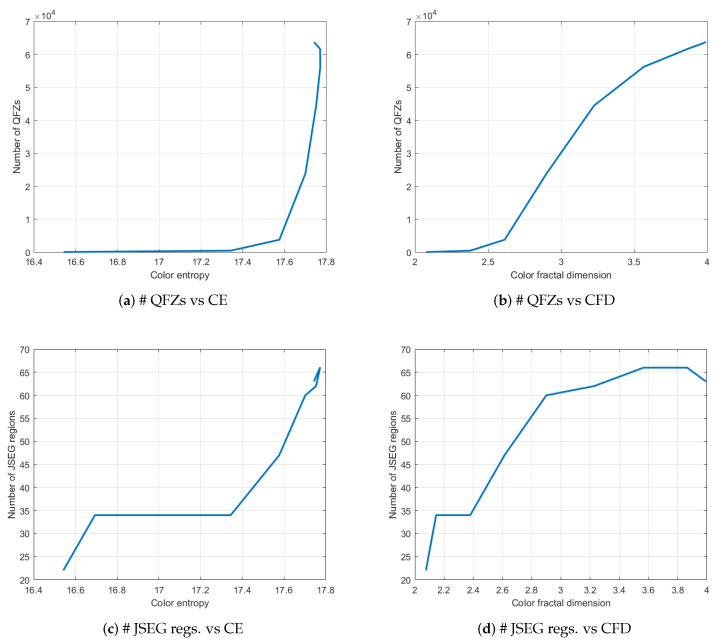
Plots of the number of QFZs and JSEG regions versus color entropy and color fractal dimension, respectively, for the fractal images in Figure 1.

In Table 1 and Figure 3 one can see that, as the fractal image complexity is increasing, the number of quasi-flat zones (# QFZs) and the number of regions segmented using JSEG increase as well, for the same choice of input parameters of the segmentation algorithms. This initial conclusion enabled the next set of experiments described in this article.

**Figure 4:**
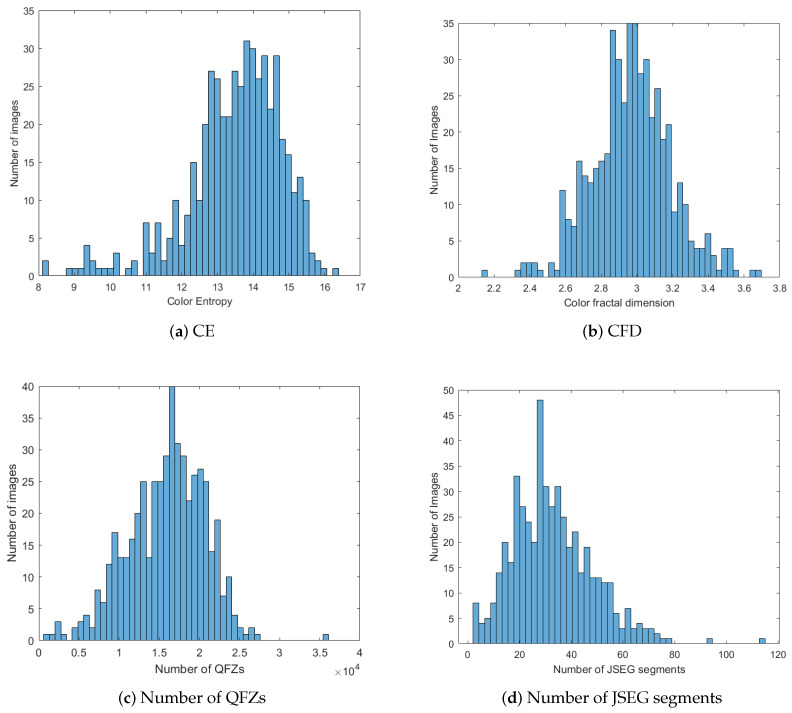
Histograms of CE, CFD, number of QFZs and number of JSEG segments on the BSDS500 data base.

Further on, we investigate the possible existing correlation between the two previously described complexity measures—color entropy and color fractal dimension—and the number of quasi-flat zones (QFZs) and number of JSEG segments, for the Berkeley image data set. We calculated the histograms of CE, CFD, number of QFZs and JSEG segments, computed on the BSDS500 data set, in order to see how all these measures are distributed over the data set. The resulting histograms are depicted in Figure 4 and it can be noticed that they exhibit more or less a Gaussian shape, characteristic to natural phenomena. In Table 2 we show the CE, CFD, number of QFZs and JSEG segments for the eight images in Figure 2. We studied the existence of a linear dependence between the image complexity, expressed as CE or CFD, and the image segmentation difficulty, expressed as number of QFZs or JSEG segments. For the computation of the correlation coefficient we used the default Matlab implementation of the Pearson, Spearman and Kendall correlation coefficient computation. In this article we report only the results obtained for the Pearson correlation, as it provides the best results compared with the other two types.

**Figure 5:**
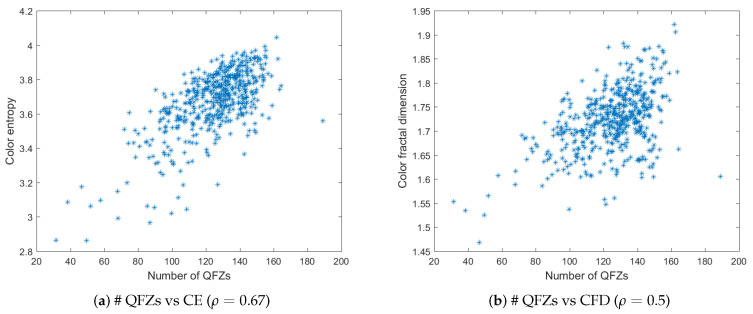
The number of QFZs versus color entropy (**a**) and color fractal dimension (**b**) for the BSDS500 data base. The corresponding Pearson correlation coefficient is given in brackets for each case.

**Figure 6:**
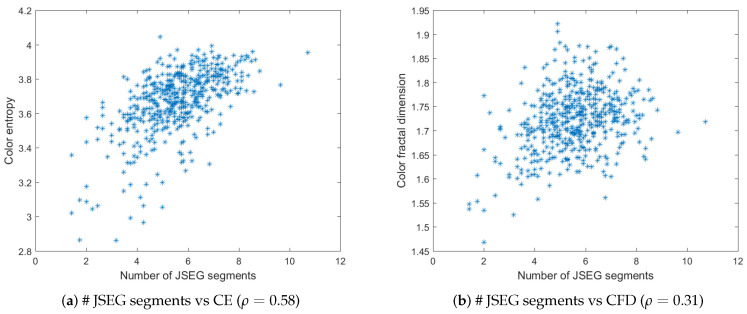
The number of JSEG segments versus color entropy (**a**) and color fractal dimension (**b**) for the BSDS500 data base. The corresponding Pearson correlation coefficient is given in brackets for each case.

**Figure 7:**
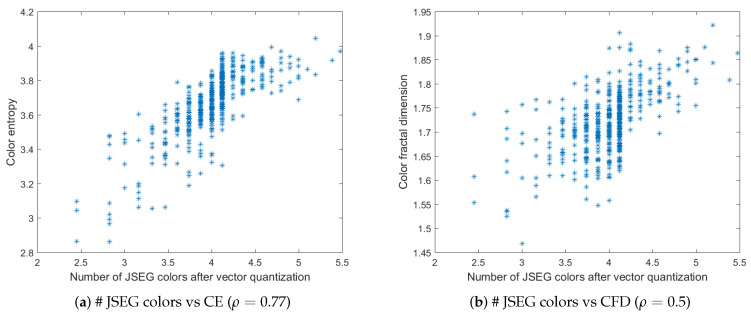
The number of JSEG colors after quantization versus color entropy (**a**) and color fractal dimension (**b**) for the BSDS500 data base. The corresponding Pearson correlation coefficient is given in brackets for each case.

Figure 5a,b depict the plots of the number of QFZs versus color entropy and color fractal dimension, respectively, computed on the BSDS500 data base. The QFZs applied on the dataset were of type $C^{\alpha ,\omega }$, with α=ω=100. Figure 6a,b depict the plots for the number of JSEG segments versus color entropy and color fractal dimension, while Figure 7a,b depict the same type of plots for the number of colors in the JSEG segmentation after color quantization. A transform of the type sqrt(z) was applied to all the data as a pre-processing step [12] in order to render a more Gaussian shape to the histograms of the data based on an analysis of the quantile-quantile plots. Also various log function-based transformations were tested, inspired from [12], in order to check if another type of correlation may apply, different from linear, but the best results are the ones reported below.

For color entropy versus the number of QFZs, the Pearson correlation coefficient ρ=0.67 indicates a considerable correlation between the two and the cloud of points tends to have an elliptical shape. For the fractal dimension versus the number of QFZs, the Pearson correlation coefficient is ρ=0.5 practically indicating there is no significant correlation, which is confirmed by the shape of the cloud of points. More or less the same observations may apply for the correlation between the color entropy and color fractal dimension on one hand and the number of JSEG segments, on the other hand, only even lower values are obtained (ρ=0.58 and ρ=0.31, respectively). Moreover, the number of reduced colors after the adaptive color quantization step in JSEG was chosen for the results in Figure 7 as this first step affects significantly the final segmentation result. As expected, there is a significant correlation between the color entropy and the number of JSEG colors (ρ=0.77) as the color entropy is defined on the initial set of colors for the image and there should be still some link to this color complexity after the color quantization step in JSEG. The Pearson correlation between CFD and the number of JSEG segments is low (ρ=0.5).

**Figure 8:**
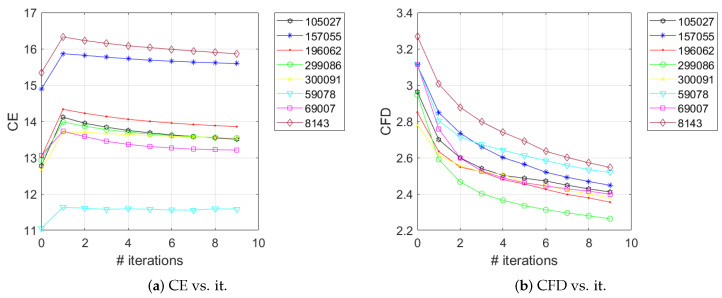
The evolution of color entropy (**a**) and color fractal dimension (**b**) as a function of the number of iterations of the Gaussian filter, for the eight images in Figure 2.

Furthermore, we wanted to assess how the variation in complexity of an image may affect the number of segments. It is known that low-pass filtering of an image reduces its complexity: e.g., blurring due to out-of-focus [49]. We are interested to quantify the drop in complexity as a consequence of low-pass filtering and to correlate the variation in complexity to the variation of the number of regions obtained by image segmentation. In our experiments we employed a low-pass Gaussian filter with a mask of size 11×11 and a standard deviation of 0.9 on the eight images in Figure 2 for several 9 iterations. Figure 8a depicts the evolution of the color entropy as a function of the number of filtering iterations. It can be noticed that, for all the eight images, there is an increase in the color entropy for the first iteration; this is caused by the fact that the filter induces additional false colors besides the original colors in the images. This increase in the number of colors naturally causes an increase in the entropy of the image. After the first iteration, the color entropy follows a decreasing tendency, as expected. The CFD curves are depicted in Figure 8b; it can be noticed that they follow a strictly decreasing tendency for all the images under consideration, more evident compared to the CE case. At the limit, as the number of low-pass filtering iterations goes to infinity, the image should become *uni* and consequently the image complexity should be null (this would correspond to CE=0 and CFD=2.0).

**Figure 9:**
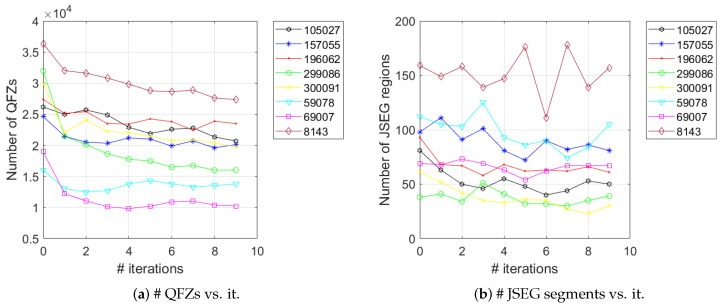
The evolution of the number of QFZs (**a**) and JSEG segments (**b**) as a function of the number of iterations of the Gaussian filter, for the eight images in Figure 2.

Figure 9 depicts the evolution of the number of QFZs and JSEG segments as a function of the number of iterations of the Gaussian filter, for the eight images in Figure 2. The evolution of the number of QFZs is smooth, clearly indicating the expected monotony, while the number of JSEG segments exhibits larger variations, though a decreasing trend can be observed.

**Figure 10:**
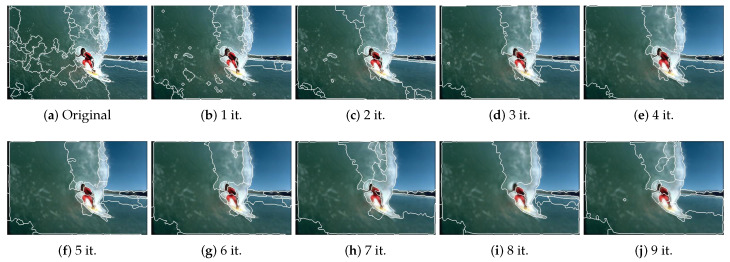
JSEG segmentation results for image 300091—the original and after applying the Gaussian filter for the specified number of iterations.

**Figure 11:**
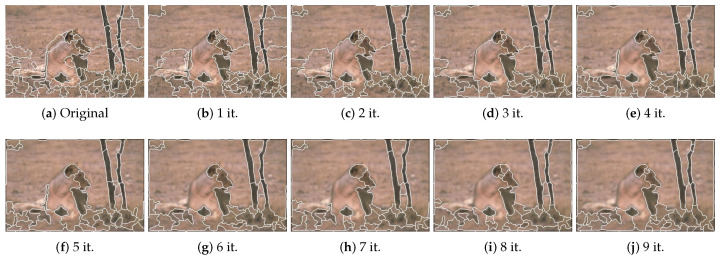
JSEG segmentation results for image 105027—the original and after applying the Gaussian filter for the specified number of iterations.

In Figure 10 and Figure 11 we show the results of JSEG image segmentation for two of the images in Figure 2 as a function of low-pass filtering iteration number. One can notice the evolution of the segmentation map, as the image complexity decreases as a consequence of low-pass filtering. As expected, the number of segments generally decreases with iterations. Though the results in Figure 9a are better supporting the correlation between image complexity measures and number of segments, the segmentation results are less relevant for showing, because of the excessive over-segmentation which is characteristic to QFZ approach.

## 4. Conclusions

In this article, we investigated the correlation between the intrinsic color image complexity and the number of segments resulting in an image over-segmentation task, for two segmentation approaches. We also analyzed how the diminishing of image complexity affects the number of segments in the resulting segmentation map. For experiments we considered two types of images: color fractal images and color natural images from the Berkeley image data base. As color image complexity measures we used the color entropy and the color fractal dimension. The embraced color image segmentation approaches were the quasi-flat zones and JSEG. The experimental results on the complete Berkeley data set showed some slight correlation between the color entropy and the number of the quasi-flat zones, while for the color fractal dimension there is no significant correlation to the number of quasi-flat zones. The same conclusion can be drawn regarding the number of JSEG segments. A more important correlation was obtained between the color entropy and the number of reduced colors after the color quantization step in JSEG. A possible explanation for the relatively low correlation between the color entropy and color fractal dimension, on one hand, and the number of QFZs and JSEG segments on the other hand, could be that the chosen complexity measures are not capable of capturing or describing the higher-level (possibly the semantic one) content of the natural images in the Berkeley data base and that for such images more appropriate complexity measures must be defined and used. The Pearson correlation coefficient values we obtained in our experiments are similar to the ones reported in [24], meaning that the image complexity and the human perception of it still require further understanding and appropriate modelling in order to better estimate the difficulty in the image segmentation task. However, the current preliminary study shows that, to some extent, one could predict the difficulty of the segmentation task based on image complexity assessment, as well as define the complexity of a color image based on the number of segments or reduce the complexity of the image by means of low-pass filtering for the purpose of reducing the burden of the segmentation task.

## Figures and Tables

**Table 1:** Color entropy, color fractal dimension, number of quasi-flat zones and number of regions segmented using JSEG for the nine color fractal images from the data set at [30].

	*H* = 0.1	*H* = 0.2	*H* = 0.3	*H* = 0.4	*H* = 0.5	*H* = 0.6	*H* = 0.7	*H* = 0.8	*H* = 0.9
CE	17.7429	17.772	17.7727	17.7532	17.7015	17.5768	17.3444	16.6938	16.5423
CFD	3.9926	3.8637	3.5655	3.2269	2.9002	2.6137	2.3791	2.1456	2.0757
# QFZs	63694	61597	56191	44503	23829	3755	437	101	20
# JSEG regs.	63	66	66	62	60	47	34	34	22

**Table 2:** Color entropy and color fractal dimension, number of quasi-flat zones and number of regions segmented using JSEG for the eight images depicted in Figure 2, from the BSDS500 data set.

Image	105027	157055	69007	299086	196062	59078	300091	8143
CE	12.7748	14.4893	13.0638	12.9328	13.0152	11.0511	12.6644	15.3425
CFD	2.9631	3.1163	3.1132	2.9468	2.9502	3.1163	2.7797	3.2685
# QFZs	26164	24641	27389	31982	29882	16025	18951	36335
# JSEG regs.	81	98	93	38	61	112	69	159
